# Supplementary material for: Sexually transmitted infections and prior antibiotic use as important causes for negative urine cultures among adults presenting with urinary tract infection symptoms to primary care clinics in Zimbabwe: a cross-sectional study
Source: BMJ Open. 2021 Aug 11;11(8):e050407. doi: 10.1136/bmjopen-2021-050407 (PMC8359455; doi:10.1136/bmjopen-2021-050407)
Supplement: Author's manuscript [file bmjopen-2021-050407.draft_revisions.pdf]

BMJ Open

BMJ Open is committed to open peer review. As part of this commitment we make the peer review history of every article we publish publicly available.

When an article is published we post the peer reviewers' comments and the authors' responses online. We also post the versions of the paper that were used during peer review. These are the versions that the peer review comments apply to.

The versions of the paper that follow are the versions that were submitted during the peer review process. They are not the versions of record or the final published versions. They should not be cited or distributed as the published version of this manuscript.

BMJ Open is an open access journal and the full, final, typeset and author-corrected version of record of the manuscript is available on our site with no access controls, subscription charges or pay-per-view fees (http://bmjopen.bmj.com).

If you have any questions on BMJ Open's open peer review process please email info.bmjopen@bmj.com

BMJ Open

Sexually transmitted infections and prior antibiotic use are important causes for negative urine cultures among adults with urinary tract infection symptoms presenting to primary care clinics in Zimbabwe

Journal:	*BMJ Open*
Manuscript ID	bmjopen-2021-050407
Article Type:	Original research
Date Submitted by the Author:	21-Feb-2021

Complete List of Authors:	Olaru, Ioana D.; London School of Hygiene & Tropical Medicine, Clinical Research Department; Biomedical Research and Training Institute, Clinical Research Department<br>Chisenga, Mutsawashe; Biomedical Research and Training Institute<br>Yeung, Shunmay; London School of Hygiene & Tropical Medicine; St Mary's Hospital, Department of Paediatric Infectious Disease<br>Mabey, David; London School of Hygiene & Tropical Medicine, Clinical Research<br>Marks, Michael; London School of Hygiene & Tropical Medicine, Clinical Research Department<br>Chonzi, Prosper; City of Harare Health Services Department, Department of Health<br>Masunda, Kudzai PE; City of Harare Health Services Department, Department of Health<br>Machiha, Anna; Ministry of Health and Child Care, AIDS and TB Unit<br>Ferrand, Rashida; London School of Hygiene and Tropical Medicine Faculty of Infectious and Tropical Diseases, Clinical Research; Biomedical Research and Training Institute<br>Kranzer, Katharina; London School of Hygiene & Tropical Medicine, Clinical Research Department; Biomedical Research and Training Institute

Keywords:	Epidemiology < INFECTIOUS DISEASES, PRIMARY CARE, SEXUAL MEDICINE, Urinary tract infections < UROLOGY

SCHOLARONE™
Manuscripts

I, the Submitting Author has the right to grant and does grant on behalf of all authors of the Work (as defined in the below author licence), an exclusive licence and/or a non-exclusive licence for contributions from authors who are: i) UK Crown employees; ii) where BMJ has agreed a CC-BY licence shall apply, and/or iii) in accordance with the terms applicable for US Federal Government officers or employees acting as part of their official duties; on a worldwide, perpetual, irrevocable, royalty-free basis to BMJ Publishing Group Ltd ("BMJ") its licensees and where the relevant Journal is co-owned by BMJ to the co-owners of the Journal, to publish the Work in this journal and any other BMJ products and to exploit all rights, as set out in our licence.

The Submitting Author accepts and understands that any supply made under these terms is made by BMJ to the Submitting Author unless you are acting as an employee on behalf of your employer or a postgraduate student of an affiliated institution which is paying any applicable article publishing charge ("APC") for Open Access articles. Where the Submitting Author wishes to make the Work available on an Open Access basis (and intends to pay the relevant APC), the terms of reuse of such Open Access shall be governed by a Creative Commons licence – details of these licences and which Creative Commons licence will apply to this Work are set out in our licence referred to above.

Other than as permitted in any relevant BMJ Author's Self Archiving Policies, I confirm this Work has not been accepted for publication elsewhere, is not being considered for publication elsewhere and does not duplicate material already published. I confirm all authors consent to publication of this Work and authorise the granting of this licence.

For peer review only

Article type: original research

Sexually transmitted infections and prior antibiotic use are important causes for negative urine cultures among adults with urinary tract infection symptoms presenting to primary care clinics in Zimbabwe

Authors: Ioana D Olaru[a,b]*, Mutsawashe Chisenga[b], Shunmay Yeung[a,c], David Mabey[a], Michael Marks[a], Prosper Chonzi[d], Kudzai PE Masunda[d], Anna Machiha[e], Rashida A Ferrand[a,b], Katharina Kranzer[a,b,f]

Affiliations

a. Clinical Research Department, London School of Hygiene and Tropical Medicine, London, United Kingdom

b. Biomedical Research and Training Institute, Harare, Zimbabwe

c. Department of Paediatric Infectious Disease, St Mary's Imperial College Hospital, London, United Kingdom

d. Department of Health, Harare City Council, Harare, Zimbabwe

e. AIDS and TB Unit, Ministry of Health and Child Care, Harare, Zimbabwe

f. Division of Infectious and Tropical Medicine, Medical Centre of the University of Munich, Munich, Germany

Correspondence to:

Ioana D Olaru:

London School of Hygiene and Tropical Medicine, Keppel Street, Bloomsbury, London WC1E 7HT, United Kingdom

Email: ioana-diana.olaru@lshtm.ac.uk

Tel: +263 77113 2503

Keywords: STI, Sexually transmitted infections, AMR, Antimicrobial use

Word count: 2304

Abstract: 253

References: 24

Tables and Figures: Tables: 2, Figures: 2

For peer review only

Abstract

Objective: Urinary tract infections (UTIs) are common in primary care. The yield of urine cultures in patients with UTI symptoms can be considerably different between high- and low-income settings. This study aimed to explore possible causes of negative urine cultures in patients presenting with symptoms of UTI to primary health clinics in Harare.

Design: Cross-sectional study

Setting: nine primary health clinics in Harare, Zimbabwe

Participants: adults presenting with symptoms of UTIs between March and July 2020

Primary outcome measures: Urine samples underwent dipstick testing, microscopy, culture, testing for sexually transmitted infections using GeneXpert and for the presence of antibiotic residues using an antibiotic bioassay. The primary outcomes were the number and proportion of participants with evidence of sexually transmitted infections, prior antibiotic exposure, leukocyturia and UTIs.

Results: The study included 425 participants with a median age of 37.3 years, of whom 275 (64.7%) were women. Leukocyturia was detected in 130 (30.6%, 95%CI 26.2-35.2) of participants and 96 (22.6%, 95%CI 18.7-26.9) had a positive urine culture for a uropathogen. *Chlamydia trachomatis, Neisseria gonorrhoeae* and *Trichomonas vaginalis* were detected in 43/425 (10.1%, 95%CI 7.4-13.4%), 37/425 (8.7%, 95%CI 6.2-11.8) and 14/175 (8.0%, 95%CI 4.4-13.1) participants respectively. Overall, 89 (20.9%, 95%CI 17.2-25.1) participants either reported having taken prior antibiotics or had a positive urine bioassay. In 170 (40.0%, 95%CI 35.3-44.8) all of the tests that were performed were negative.

Conclusions: This study found a high prevalence of sexually transmitted infections and evidence of prior antimicrobial use as possible explanations for the low proportion of positive urine cultures.

Strengths and limitations of this study

- This is the first study exploring the causes for negative urine cultures among adults presenting with urinary tract infection symptoms to primary care in a low-income setting.
- The findings of this study highlight the high prevalence of sexually transmitted infections and the need for diagnostic testing to ensure appropriate treatment.

For peer review only - http://bmjopen.bmj.com/site/about/guidelines.xhtml

- By using two different methods the study showed a high prevalence of prior antibiotic exposure which should prompt improvements of regulations around antibiotic access.
- Testing for sexually transmitted infections was limited to three major pathogens which may have led to an underestimation of the prevalence of sexually transmitted infections.
- The prevalence of sexually transmitted infections is not generalisable to all primary health care attendees, as the participants were enrolled if they reported the presence of urinary tract infection symptoms.

For peer review only

For peer review only - http://bmjopen.bmj.com/site/about/guidelines.xhtml

Introduction

Urinary tract infections (UTIs) are a common reason for presentation to primary care with 10% of adult women experiencing at least one episode per year.[1] Dysuria, which is one of the most common symptoms suggestive of a UTI, is reported in 2-5% of presentations in general practice.[2] While dysuria and other urinary symptoms such as frequency and urgency may be attributed to acute bacterial cystitis, they are by no means specific.[3] Symptoms can be due to a wide range of infectious agents as well as to other non-infectious causes (Table 1). In high-income settings acute bacterial cystitis is the most common cause of UTI symptoms in women presenting to primary care.[2] Thus the symptom complex is treated empirically with antibiotics and diagnostic tests are not deemed routinely necessary. Patients whose symptoms do not respond to first-line antibiotics are reassessed for other diagnoses or infections with organisms resistant to first-line antibiotics.[4]

Table 1. Causes of symptoms with acute onset that may be suggestive of a urinary tract infection in adults

**Category**		**Specific cause**
**Infectious**	Acute bacterial cystitis	*Escherichia coli*<br>*Klebsiella pneumoniae*<br>*Staphylococcus saprophyticus*<br>*Enterococcus spp.*<br>Unidentified (partially treated)
	Prostatitis	*Escherichia coli*<br>*Klebsiella pneumoniae*<br>*Proteus mirabilis*<br>*Pseudomonas aeruginosa*<br>*Enterococcus spp.*
	Sexually transmitted infections	*Chlamydia trachomatis*<br>*Neisseria gonorrhoea*<br>*Trichomonas vaginalis*<br>*Mycoplasma hominis*<br>*Ureaplasma urealyticum*<br>*Herpes simplex*<br>*Candida spp.*
	Other infections	*Mycobacterium tuberculosis* (urogenital)<br>*Schistosoma haematobium*
**Inflammatory**	Dermatologic	Irritant or contact dermatitis<br>Stevens-Johnson syndrome
	Non-infectious	Foreign body (e.g. stone)<br>Reactive arthritis with urethritis
**Non-inflammatory**	Drug or food related	Spermicides, topical hygiene products, drugs, certain foods
	Traumatic	Urogenital instrumentation/ surgery<br>Horseback or bicycle riding

For peer review only

For peer review only - http://bmjopen.bmj.com/site/about/guidelines.xhtml

The prevalence of positive urine cultures in patients presenting with presumed UTIs varies widely across different settings. This is particularly true when comparing studies from high- and low-income settings.[2, 5] Possible explanations include i) differences in prevalence of other conditions causing similar symptoms; ii) unregulated access to antibiotics leading to partially treated infections; or to iii) differences in laboratory techniques affecting the limit of detection.

This study aimed to evaluate possible causes of negative urine cultures in patients presenting with symptoms of UTI to primary health clinics in Harare.

Methods

Adults (aged 18 years and above) presenting with symptoms of UTIs to nine primary health clinics in Harare were enrolled into the ARGUS study (Antimicrobial Resistance in Gram-negatives from Urinary Specimens). In Zimbabwe, primary health clinics are nurse-led and serve the population living in their catchment area. Patients accessing the acute medical services have to pay a user fee. Access to diagnostic tests is limited beyond HIV, tuberculosis and malaria. The eligibility criteria and enrolment procedures for the ARGUS study were previously described.[6] Patients were included into the study if they presented with at least two symptoms suggestive of UTI (dysuria, urgency, frequency, suprapubic pain and/or flank pain).

This is a cross-sectional study reporting on a subset of participants from the ARGUS study who were consecutively enrolled between March and July 2020. Mid-stream urine samples were collected from study participants and underwent dipstick testing, microscopy and culture using conventional microbiology methods.[6] Leukocyturia was considered to be present if the sample had ≥10 white blood cells/µl on microscopy or a positive dipstick for leucocytes. Antibacterial activity of urine indicating possible prior antibiotic exposure was ascertained using a urine bioassay. Briefly, 20 µl urine were inoculated on a 6-mm filter paper disc that was incubated on Muller-Hinton and using *E. coli (*ATCC25922) as an indicator organism.[7] Antibacterial activity was considered to be present in the sample if a growth inhibition zone around the disc was observed following incubation for 24 hours at 37°C.

Urine samples were tested for sexually transmitted infections (STIs), specifically *Chlamydia trachomatis* and *Neisseria gonorrhoea*. For women only, urine samples were also tested for

For peer review only - http://bmjopen.bmj.com/site/about/guidelines.xhtml

Trichomonas vaginalis. STI testing was done using the GeneXpert platform (Cepheid, Sunnyvale, CA) according to the manufacturer's instructions. Treatment for STIs was prescribed during the initial visit by the clinic nurse according to routine practice and independent of study procedures. Participants with positive tests were notified of their result and offered treatment for themselves and their partners if they were not treated on their initial appointment. To ascertain if symptoms had resolved, a follow-up phone call was conducted 28 days post enrolment.

Statistical analysis

Data were analysed using STATA v.15 (StataCorp, TX, USA). Categorical data were described using counts and percentages and continuous data using medians and interquartile ranges (IQRs). Differences between categorical and continuous variables were evaluated using the chi-square and Mann Whitney U tests, respectively. The level of significance was set at $p \leq 0.05$.

The study was approved by the London School of Hygiene and Tropical Medicine ethics committee (Ref. 16424) and the Medical Research Council of Zimbabwe (MRCZ/A/2406). All study participants provided written informed consent.

Patient and public involvement

None in the study design. Results from this study were disseminated to nurses working in the polyclinics from where the study participants were recruited in order to improve patient care.

Results

The study included 425 participants with a median age of 37.3 years (IQR 26.8-48.0), of whom 275 (64.7%) were women and 172 (40.5%) were HIV-positive. All samples were tested for the presence of leukocytes, underwent conventional urine culture and were tested for *C. trachomatis* and *N. gonorrhoea*. Samples from 175 women were tested for *T. vaginalis*. Of 425 urine samples collected, 420 were stored for the antibiotic bioassay; volume was insufficient for storage for the remaining five samples. In 255 (60.0%) of participants at least one of the tests was positive while in 170 (40.0%) all of the tests that were performed were negative and the cause of their symptoms could not be determined or suspected. Figure 1 shows the distribution of positive test results.

Leukocyturia was detected in 130 (30.6%) of participants while two (0.5%) had haematuria. Urine cultures were positive in 96 (22.6%), negative in 314 (73.9%) and contaminated in 15

For peer review only - http://bmjopen.bmj.com/site/about/guidelines.xhtml

For peer review only

(3.5%) participants. A higher proportion of urine cultures were positive in women (n=73; 26.6%) compared to men (n=23, 15.3%). Urine culture positivity was higher in participants with more recent symptom onset prior to presentation (58/225, 25.8% in those with a symptom onset of ≤7 days, vs. 37/197, 18.8% for those with symptoms >7 days, p=0.086).

Overall, 83/425 (19.5%, 95%CI 15.9-23.6) participants had an STI including 11 who also had a positive urine culture. The prevalence of *C. trachomatis* was 43/425 (10.1%, 95%CI 7.4-13.4%), of *N. gonorrhoeae* 37/425 (8.7%, 95%CI 6.2-11.8) and of *T. vaginalis* 14/175 (8.0%, 95%CI 4.4-13.1). Eleven (2.6%) participants had a positive STI test for two pathogens. The prevalence of *C. trachomatis* was 26/275 (9.5% 95%CI 6.3-13.5) among women and 17/133 (12.8% 95%CI 7.6-19.7) among men. For *N. gonorrhoea* prevalence was 18/275 (6.5% 95%CI 3.6-10.1) among women and 19/150 (12.7% 95%CI 7.8-19.1, p=0.032) among men. STIs were more common in those with recent symptom onset (52/225, 23.1% among those with an onset of symptoms of ≤7 days and 31/197, 15.7% for those with symptoms >7 days, p =0.057). Participants who tested positive for an STI, were younger and were less likely to have HIV-infection than those without an STI (Table 2).

Table 2. Characteristics of study participants according to test result

**Characteristic**	**Total N=425 (%)**	**STI positive N=83 (%)**	**Urine culture positive N=96 (%)**	**Leukocyturia N=130 (%)**	**Prior antibiotics N=89 (%)**	**All tests negative N=170 (%)**
Age, median (IQR)	37.3 (26.8-48.0)	28.4 (23.5-39.2)	39.8 (26.0-50.0)	34.2 (24.8-47.0)	37.6 (27.6-49.4)	39.4 (29.6-49.6)
Female sex	275 (64.7)	51 (61.5)	73 (76.0)	88 (67.7)	51 (57.3)	107 (62.9)
Completed at least secondary education	359 (84.5)	71 (85.5)	75 (78.1)	108 (83.1)	77 (86.5)	141 (82.9)
HIV positive*	172 (42.4)	20 (25.6)	42 (47.2)	53 (43.4)	38 (44.2)	76 (46.1)
Pregnancy*	35 (13.0)	4 (7.8)	7 (9.7)	15 (17.2)	5 (9.8)	16 (15.5)
Married/ have a partner	325 (76.5)	66 (79.5)	74 (77.1)	91 (70.0)	67 (75.3)	132 (77.7)
Duration of symptoms prior to presentation	7 (5-12)	7 (4-12)	7 (4-11)	7 (5-10)	7 (5-12)	9 (6-12)
Symptoms resolved on follow-up*	334 (85.0)	70 (92.1)	74 (83.2)	105 (89.0)	73 (89.0)	127 (79.9)

For peer review only - http://bmjopen.bmj.com/site/about/guidelines.xhtml

**19 participants did not know their HIV status; 5 women did not know if they were pregnant; follow-up information available for 393 participants.*
IQR: interquartile range; STI: sexually transmitted infection

The median duration of symptoms was 7 days for participants with positive urine cultures and those who had an STI. Among participants who did not have an STI or a positive urine culture, the median duration of symptoms was 8 days (IQR 5-12). The most common presenting symptoms were dysuria (337/425, 79.3%), frequency (310/425, 72.9%), and suprapubic pain (272/425, 64.0%). Overall presenting symptoms were similar between individuals with STIs, UTIs and those with negative cultures (Figure 2).

Antimicrobial treatment prior to clinic presentation was reported by 30 (7.1%) while the antibiotic bioassay was positive in 73 (17.4%) including 26 participants with HIV infection who were taking co-trimoxazole prophylaxis. Overall, 89 (20.9%) of participants had either reported taking antibiotics or had a positive urine bioassay.

STI treatment was prescribed by the clinic nurses at the initial visit in 38 participants, of whom 20 (53%) had a positive STI test while 63 (76%) of the 83 patients who had an STI were not prescribed STI treatment by the primary health care nurse.

A follow up phone call was made with 393 (92.5%) of the participants. Most (334/393, 85.0%) reported that the symptoms had resolved or significantly improved since enrolment. Among the 59 participants whose symptoms did not improve or resolve, 15 had a positive urine culture and six had an STI while 32 (54.2%) did not have any positive tests. Symptom resolution or improvement was reported by 70/76 (92.1%) participants with an STI, by 74/89 (83.1%) of those with a positive urine culture and by 127/159 (79.9%) with negative results in all tests. Among participants with positive urine cultures, symptoms improved or resolved in 64/67 (95.5%) of those who reported having taken their prescribed treatment and in 9/20 (45%) of those who had not.

Discussion

This study found that only one in five people presenting to primary care in Harare with UTI symptoms had a positive urine culture with a uropathogen explaining their presenting symptoms. There was a high prevalence of STIs and evidence of prior antimicrobial use among

For peer review only - http://bmjopen.bmj.com/site/about/guidelines.xhtml

study participants as possible explanations for the low proportion of positive urine cultures. However, in 40% of patients, symptoms remained unexplained.

Only 20% of study participants had a positive urine culture which is similar to findings from studies conducted in other sub-Saharan African countries where culture positivity was found to be 11% and 36% among outpatients.[5, 8] In contrast, studies conducted among women with cystitis in high-income countries mostly from Europe and the Americas, report that 75-80% of urine cultures reveal significant growth of uropathogens.[2, 9, 10] This may be explained by differences in access to healthcare, unregulated antibiotic use and prevalence of other conditions with similar symptoms between settings.[5, 11-13]

In our study, the median time to presentation was 7 days while studies from high-income settings report a much shorter time between symptom onset and clinic presentation of 2-3 days.[9, 10] Thus, the lower yield of urine cultures in this study may be due to spontaneous resolution of infection because of the delays in accessing healthcare or self-treatment with antibiotics.[14] This may particularly be the case in patients with leukocyturia where symptoms can be explained by residual inflammation. Dysuria and leukocyturia in the absence of positive urine cultures may be due to other causes such as urogenital tuberculosis, schistosomiasis and other inflammatory conditions.[3] However, other infections are unlikely to be the cause of symptoms in our patient population given that for the vast majority, the symptoms resolved or improved on follow-up and that haematuria was present in less than 1%.

Antimicrobial use prior to clinic presentation may also have contributed to a lower urine culture yield with one in five study participants having evidence of taking antibiotics prior to presentation to primary care. While only 7% of the study participants reported antimicrobial use in the previous two weeks, 17% had evidence of antimicrobial activity in their urine. Although in Zimbabwe antibiotic access from pharmacies is highly regulated, antibiotics can be acquired from the informal market.[15] Patients may not report on self-treatment at clinic presentation due to social desirability bias. Prior antimicrobial use was similar to that reported by patients from Senegal [16] which had a culture positivity of 27% but lower than in studies from the United States.[17] This suggests that asking patients about antimicrobials may not be sufficient in determining prior exposure as shown in findings from Ghana.[18] On the other hand the antibiotic bioassay may overestimate antimicrobial use for treatment of infections by also detecting antimicrobials consumed unintentionally in food and water.[19] Alternative

For peer review only - http://bmjopen.bmj.com/site/about/guidelines.xhtml

For peer review only

explanations for symptoms suggestive of UTIs such as dysuria include irritant hygienic products, spermicides and certain foods.[3]

STIs are highly prevalent in some sub-Saharan African setting[20] and there is some symptom overlap between STIs and UTIs. A meta-analysis of studies from sub-Saharan Africa reported a pooled prevalence for *C. trachomatis* of 7.8% in women of reproductive age[21] while a study from Zimbabwe testing mainly asymptomatic adolescents and young people found a prevalence of 17% of *C. trachomatis* and/or *Neisseria gonorrhoea*.[22] While STIs may be asymptomatic particularly in women, patients with symptoms due to STIs frequently experience diagnostic and treatment delays leading to prolonged symptoms prior to presentation.[23] One of the study eligibility criteria included recent symptom onset (within two weeks). This was aimed at increasing the likelihood of UTIs compared to STIs. Despite this, STI prevalence in our study was almost 20%. Importantly one in fifteen women and one in eight men tested positive for *Neisseria gonorrhoea*. The high prevalence of STIs may also explain the large proportion of men enrolled into the study, who are generally more likely to experience symptoms from STIs, which are similar to symptoms of UTIs.[23] The study may have missed other STIs because testing was limited to three major pathogens but did not include other organisms that may cause urethritis and urinary tract symptoms such as *Mycoplasma genitalium* leading to an underestimation of STI prevalence. Furthermore, STIs may have been missed because the study used mid-stream urine samples while first-catch samples are recommended for the diagnosis of STIs for optimal yield on urine samples. Genital ulcers caused by herpes simplex and vaginitis due to candida may explain symptoms in some patients. Also, men were not tested for *T. vaginalis* as this is less frequent in men.[24]

Only a quarter of participants who tested positive for an STI, received STI treatment on the initial clinic presentation because the attending nurse did not suspect an STI. This highlights the difficulties in differentiating the two conditions on clinical grounds. Access to diagnostic testing at primary care level is thus urgently needed.

The prevalence of STIs is not generalisable to all primary health care attendees, as the participants were selected on the basis of STI/UTI symptoms.

Conclusions

For peer review only - http://bmjopen.bmj.com/site/about/guidelines.xhtml

This is the first study exploring the causes for negative urine cultures among adults presenting with UTI symptoms to primary care in a low-income setting. The findings of this study highlight the high STI burden in this patient population. The overlap in symptoms in patients diagnosed with UTIs and STIs respectively results in underdiagnosis and undertreatment of STIs. Diagnostic testing is paramount to ensure appropriate treatment. Very few participants who had an STI received appropriate treatment on clinic presentation because STIs were not suspected emphasizing the missed opportunities for diagnosis and treatment. The high prevalence of prior antibiotic exposure in patients presenting to primary care should prompt an improvement of regulations around antibiotic access to prevent development of antimicrobial resistance.

Funding

IDO received funding though the Wellcome Trust Clinical PhD Programme awarded to the London School of Hygiene & Tropical Medicine (grant number 203905/Z/16/Z). The funders had no role in study design, data collection and interpretation, or the decision to submit the work for publication.

Competing interests: none declared.

Ethical approval: The study was approved by the London School of Hygiene and Tropical Medicine ethics committee (Ref. 16424) and the Medical Research Council of Zimbabwe (MRCZ/A/2406). All study participants provided written informed consent.

Data availability statement: the data underlying this study can be obtained upon request by emailing the corresponding author.

Author contributions: IDO and KK conceived the study idea and planned the study. RAF contributed to the study idea. PC, KPEM and AM provided support with the resources needed for the study. SY and RAF provided support with conducting the study. IDO and MC conducted the laboratory testing and interpretation of results. IDO and KK analysed and interpreted the data, and drafted the initial manuscript. MC, SY, DM, MM, PC, KPE, AM, and RF reviewed and provided feedback on the initial manuscript. All authors read and approved the final manuscript.

For peer review only - http://bmjopen.bmj.com/site/about/guidelines.xhtml

Figure 1. Distribution of positive tests among study participants (170/425 samples, 40% were negative for all tests)

Figure 2. Presenting symptoms in study participants according to urine culture and STI test result

For peer review only

For peer review only - http://bmjopen.bmj.com/site/about/guidelines.xhtml

References

1. Foxman B, Barlow R, D'Arcy H, Gillespie B, Sobel JD. Urinary tract infection: self-reported incidence and associated costs. Ann Epidemiol. 2000;10(8):509-15. Epub 2000/12/19. doi: 10.1016/s1047-2797(00)00072-7. PubMed PMID: 11118930.

2. Heytens S, Boelens J, Claeys G, DeSutter A, Christiaens T. Uropathogen distribution and antimicrobial susceptibility in uncomplicated cystitis in Belgium, a high antibiotics prescribing country: 20-year surveillance. Eur J Clin Microbiol Infect Dis. 2017;36(1):105-13. Epub 2016/09/19. doi: 10.1007/s10096-016-2776-8. PubMed PMID: 27639858.

3. Michels TC, Sands JE. Dysuria: Evaluation and Differential Diagnosis in Adults. Am Fam Physician. 2015;92(9):778-86. Epub 2015/11/12. PubMed PMID: 26554471.

4. World Health Organization. IMAI district clinician manual: hospital care for adolescents and adults: guidelines for the management of illnesses with limited-resources. WHO, Geneva, 2011. Available from https://apps.who.int/iris/bitstream/handle/10665/77751/9789241548281_Vol1_eng.pdf.

5. Iregbu K.C., P.I. N-P. Urinary tract infections in a tertiary hospital in Abuja, Nigeria. African Journal of Clinical and Experimental Microbiology. 2013;14:169-73.

6. Olaru ID, Yeung S, Ferrand RA, Stabler R, Chonzi P, Mabey D, et al. Antimicrobial Resistance in Gram-negative bacteria from Urinary Specimens: a study of prevalence, risk factors and molecular mechanisms of resistance (ARGUS) in Zimbabwe - a study protocol. Wellcome Open Res. 2020;5:140. Epub 2020/10/23. doi: 0.12688/wellcomeopenres.15977.1. PubMed PMID: 33088923; PubMed Central PMCID: PMCPMC7551514.

7. Driscoll AJ, Bhat N, Karron RA, O'Brien KL, Murdoch DR. Disk diffusion bioassays for the detection of antibiotic activity in body fluids: applications for the Pneumonia Etiology Research for Child Health project. Clin Infect Dis. 2012;54 Suppl 2:S159-64. Epub 2012/03/21. doi: 10.1093/cid/cir1061. PubMed PMID: 22403231.

8. Bitew A, Molalign T, Chanie M. Species distribution and antibiotic susceptibility profile of bacterial uropathogens among patients complaining urinary tract infections. BMC Infect Dis. 2017;17(1):654. Epub 2017/10/01. doi: 10.1186/s12879-017-2743-8. PubMed PMID: 28962545; PubMed Central PMCID: PMCPMC5622472.

9. Hooton TM, Roberts PL, Cox ME, Stapleton AE. Voided midstream urine culture and acute cystitis in premenopausal women. N Engl J Med. 2013;369(20):1883-91. Epub 2013/11/15. doi: 10.1056/NEJMoa1302186. PubMed PMID: 24224622; PubMed Central PMCID: PMCPMC4041367.

For peer review only - http://bmjopen.bmj.com/site/about/guidelines.xhtml

10. Naber KG, Schito G, Botto H, Palou J, Mazzei T. Surveillance study in Europe and Brazil on clinical aspects and Antimicrobial Resistance Epidemiology in Females with Cystitis (ARESC): implications for empiric therapy. Eur Urol. 2008;54(5):1164-75. Epub 2008/05/31. doi: 10.1016/j.eururo.2008.05.010. PubMed PMID: 18511178.
11. Auta A, Hadi MA, Oga E, Adewuyi EO, Abdu-Aguye SN, Adeloye D, et al. Global access to antibiotics without prescription in community pharmacies: A systematic review and meta-analysis. J Infect. 2019;78(1):8-18. Epub 2018/07/10. doi: 10.1016/j.jinf.2018.07.001. PubMed PMID: 29981773.
12. Bichler KH, Savatovsky I, Members of the Urinary Tract Infection Working Group of the Guidelines Office of the European Association of U, Naber KG, Bischop MC, Bjerklund-Johansen TE, et al. EAU guidelines for the management of urogenital schistosomiasis. Eur Urol. 2006;49(6):998-1003. Epub 2006/03/08. doi: 10.1016/j.eururo.2006.02.022. PubMed PMID: 16519990.
13. Muneer A, Macrae B, Krishnamoorthy S, Zumla A. Urogenital tuberculosis - epidemiology, pathogenesis and clinical features. Nat Rev Urol. 2019;16(10):573-98. Epub 2019/09/25. doi: 10.1038/s41585-019-0228-9. PubMed PMID: 31548730.
14. Hoffmann T, Peiris R, Mar CD, Cleo G, Glasziou P. Natural history of uncomplicated urinary tract infection without antibiotics: a systematic review. Br J Gen Pract. 2020;70(699):e714-e22. Epub 2020/09/23. doi: 10.3399/bjgp20X712781. PubMed PMID: 32958533; PubMed Central PMCID: PMCPMC7510849.
15. EUCAST guidelines for detection of resistance mechanisms and specific resistances of clinical and/or epidemiological importance Version 2.01 July 2017. Available from https://eucast.org/resistance_mechanisms/.
16. Barry MS, Diallo BA, Kante D, Diallo IS. Antimicrobial susceptibility profile of community-acquired urinary tract infection in adults: A seven months prospective cross-sectional study in Dakar Town, Senegal. African Journal of Urology. 2017;23(2):166-71.
17. Talan DA, Krishnadasan A, Abrahamian FM, Stamm WE, Moran GJ, Group EMINS. Prevalence and risk factor analysis of trimethoprim-sulfamethoxazole- and fluoroquinolone-resistant Escherichia coli infection among emergency department patients with pyelonephritis. Clin Infect Dis. 2008;47(9):1150-8. Epub 2008/09/24. doi: 10.1086/592250. PubMed PMID: 18808361.
18. Donkor GY, Dontoh E, Owusu-Ofori A. A cross-sectional study on the prevalence of antibiotic use prior to laboratory tests at two Ghanaian hospitals. PLoS One.

For peer review only - http://bmjopen.bmj.com/site/about/guidelines.xhtml

2019;14(1):e0210716. Epub 2019/01/16. doi: 10.1371/journal.pone.0210716. PubMed PMID: 30645626; PubMed Central PMCID: PMCPMC6333348.

19. Azanu D, Styrishave B, Darko G, Weisser JJ, Abaidoo RC. Occurrence and risk assessment of antibiotics in water and lettuce in Ghana. Sci Total Environ. 2018;622-623:293-305. Epub 2017/12/08. doi: 10.1016/j.scitotenv.2017.11.287. PubMed PMID: 29216470.

20. Rowley J, Vander Hoorn S, Korenromp E, Low N, Unemo M, Abu-Raddad LJ, et al. Chlamydia, gonorrhoea, trichomoniasis and syphilis: global prevalence and incidence estimates, 2016. Bull World Health Organ. 2019;97(8):548-62P. Epub 2019/08/07. doi: 10.2471/BLT.18.228486. PubMed PMID: 31384073; PubMed Central PMCID: PMCPMC6653813.

21. Hussen S, Wachamo D, Yohannes Z, Tadesse E. Prevalence of chlamydia trachomatis infection among reproductive age women in sub Saharan Africa: a systematic review and meta-analysis. BMC Infect Dis. 2018;18(1):596. Epub 2018/11/28. doi: 10.1186/s12879-018-3477-y. PubMed PMID: 30477441; PubMed Central PMCID: PMCPMC6258386.

22. Martin K, Olaru ID, Buwu N, Bandason T, Marks M, Dauya E, et al. Uptake of and factors associated with testing for sexually transmitted infections in community-based settings among youth in Zimbabwe: a mixed-methods study. Lancet Child Adolesc Health. 2021;5(2):122-32. Epub 2021/01/09. doi: 10.1016/S2352-4642(20)30335-7. PubMed PMID: 33417838.

23. Hoffman CM, Fritz L, Matlakala N, Mbambazela N, Railton JP, McIntyre JA, et al. Community-based strategies to identify the unmet need for care of individuals with sexually transmitted infection-associated symptoms in rural South Africa. Trop Med Int Health. 2019;24(8):987-93. Epub 2019/05/30. doi: 10.1111/tmi.13274. PubMed PMID: 31141301.

24. Chitneni P, Beksinska M, Dietrich JJ, Jaggernath M, Closson K, Smith P, et al. Partner notification and treatment outcomes among South African adolescents and young adults diagnosed with a sexually transmitted infection via laboratory-based screening. Int J STD AIDS. 2020;31(7):627-36. Epub 2020/05/15. doi: 10.1177/0956462420915395. PubMed PMID: 32403988; PubMed Central PMCID: PMCPMC7357572.

For peer review only

For peer review only - http://bmjopen.bmj.com/site/about/guidelines.xhtml

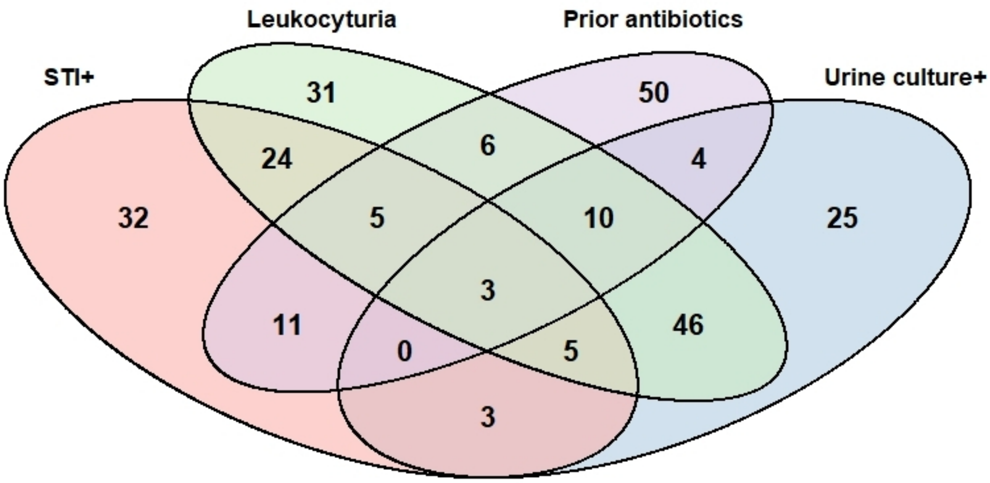

64x38mm (300 x 300 DPI)

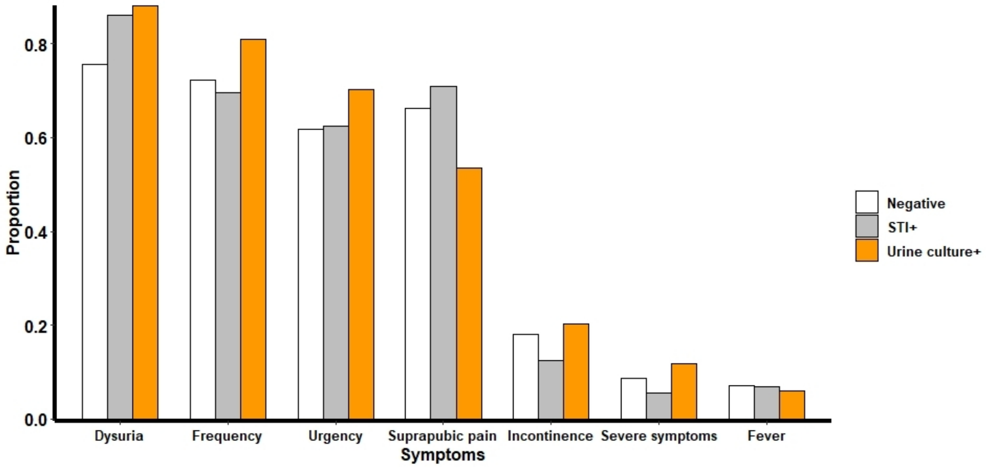

80x38mm (300 x 300 DPI)

BMJ Open

Sexually transmitted infections and prior antibiotic use as important causes for negative urine cultures among adults presenting with urinary tract infection symptoms to primary care clinics in Zimbabwe, a cross-sectional study

Journal:	*BMJ Open*
Manuscript ID	bmjopen-2021-050407.R1
Article Type:	Original research
Date Submitted by the Author:	05-Jul-2021
Complete List of Authors:	Olaru, Ioana D.; London School of Hygiene & Tropical Medicine, Clinical Research Department; Biomedical Research and Training Institute, Clinical Research Department<br>Chisenga, Mutsawashe; Biomedical Research and Training Institute<br>Yeung, Shunmay; London School of Hygiene & Tropical Medicine; St Mary's Hospital, Department of Paediatric Infectious Disease<br>Mabey, David; London School of Hygiene & Tropical Medicine, Clinical Research<br>Marks, Michael; London School of Hygiene & Tropical Medicine, Clinical Research Department<br>Chonzi, Prosper; City of Harare Health Services Department, Department of Health<br>Masunda, Kudzai PE; City of Harare Health Services Department, Department of Health<br>Machiha, Anna; Ministry of Health and Child Care, AIDS and TB Unit<br>Ferrand, Rashida; London School of Hygiene and Tropical Medicine Faculty of Infectious and Tropical Diseases, Clinical Research; Biomedical Research and Training Institute<br>Kranzer, Katharina; London School of Hygiene & Tropical Medicine, Clinical Research Department; Biomedical Research and Training Institute
**Primary Subject Heading**:	Infectious diseases
Secondary Subject Heading:	Epidemiology, General practice / Family practice, Global health, Sexual health
Keywords:	Epidemiology < INFECTIOUS DISEASES, PRIMARY CARE, SEXUAL MEDICINE, Urinary tract infections < UROLOGY

SCHOLARONE™
Manuscripts

I, the Submitting Author has the right to grant and does grant on behalf of all authors of the Work (as defined in the below author licence), an exclusive licence and/or a non-exclusive licence for contributions from authors who are: i) UK Crown employees; ii) where BMJ has agreed a CC-BY licence shall apply, and/or iii) in accordance with the terms applicable for US Federal Government officers or employees acting as part of their official duties; on a worldwide, perpetual, irrevocable, royalty-free basis to BMJ Publishing Group Ltd ("BMJ") its licensees and where the relevant Journal is co-owned by BMJ to the co-owners of the Journal, to publish the Work in this journal and any other BMJ products and to exploit all rights, as set out in our licence.

The Submitting Author accepts and understands that any supply made under these terms is made by BMJ to the Submitting Author unless you are acting as an employee on behalf of your employer or a postgraduate student of an affiliated institution which is paying any applicable article publishing charge ("APC") for Open Access articles. Where the Submitting Author wishes to make the Work available on an Open Access basis (and intends to pay the relevant APC), the terms of reuse of such Open Access shall be governed by a Creative Commons licence – details of these licences and which Creative Commons licence will apply to this Work are set out in our licence referred to above.

Other than as permitted in any relevant BMJ Author's Self Archiving Policies, I confirm this Work has not been accepted for publication elsewhere, is not being considered for publication elsewhere and does not duplicate material already published. I confirm all authors consent to publication of this Work and authorise the granting of this licence.

For peer review only

Article type: original research

Sexually transmitted infections and prior antibiotic use as important causes for negative urine cultures among adults presenting with urinary tract infection symptoms to primary care clinics in Zimbabwe, a cross-sectional study

Authors: Ioana D Olaru[a,b]*, Mutsawashe Chisenga[b], Shunmay Yeung[a,c], David Mabey[a], Michael Marks[a], Prosper Chonzi[d], Kudzai PE Masunda[d], Anna Machiha[e], Rashida A Ferrand[a,b], Katharina Kranzer[a,b,f]

Affiliations

[a] Clinical Research Department, London School of Hygiene and Tropical Medicine, London, United Kingdom

[b] Biomedical Research and Training Institute, Harare, Zimbabwe

[c] Department of Paediatric Infectious Disease, St Mary's Imperial College Hospital, London, United Kingdom

[d] Department of Health, Harare City Council, Harare, Zimbabwe

[e] AIDS and TB Unit, Ministry of Health and Child Care, Harare, Zimbabwe

[f] Division of Infectious and Tropical Medicine, Medical Centre of the University of Munich, Munich, Germany

Correspondence to:

Ioana D Olaru:

London School of Hygiene and Tropical Medicine, Keppel Street, Bloomsbury, London WC1E 7HT, United Kingdom

Email: ioana-diana.olaru@lshtm.ac.uk

Tel: +263 77113 2503

Keywords: STI, Sexually transmitted infections, AMR, Antimicrobial use

Word count: 2304

Abstract: 253

References: 24

Tables and Figures: Tables: 2, Figures: 2

For peer review only

Abstract

Objective: Urinary tract infections (UTIs) are common in primary care. The yield of urine cultures in patients with UTI symptoms can be considerably different between high- and low-income settings. This study aimed to explore possible causes of negative urine cultures in patients presenting with symptoms of UTI to primary health clinics in Harare.

Design: Cross-sectional study

Setting: nine primary health clinics in Harare, Zimbabwe

Participants: adults presenting with symptoms of UTIs between March and July 2020

Primary outcome measures: Urine samples underwent dipstick testing, microscopy, culture, testing for sexually transmitted infections using GeneXpert and for the presence of antibiotic residues using an antibiotic bioassay. The primary outcomes were the number and proportion of participants with evidence of sexually transmitted infections, prior antibiotic exposure, leukocyturia and UTIs.

Results: The study included 425 participants with a median age of 37.3 years, of whom 275 (64.7%) were women. Leukocyturia was detected in 130 (30.6%, 95%CI 26.2-35.2) of participants and 96 (22.6%, 95%CI 18.7-26.9) had a positive urine culture for a uropathogen. *Chlamydia trachomatis, Neisseria gonorrhoeae* and *Trichomonas vaginalis* were detected in 43/425 (10.1%, 95%CI 7.4-13.4%), 37/425 (8.7%, 95%CI 6.2-11.8) and 14/175 (8.0%, 95%CI 4.4-13.1) participants respectively. Overall, 89 (20.9%, 95%CI 17.2-25.1) participants either reported having taken prior antibiotics or had a positive urine bioassay. In 170 (40.0%, 95%CI 35.3-44.8) all of the tests that were performed were negative.

Conclusions: This study found a high prevalence of sexually transmitted infections and evidence of prior antimicrobial use as possible explanations for the low proportion of positive urine cultures.

Strengths and limitations of this study

- This is the first study exploring the causes for negative urine cultures among adults presenting with urinary tract infection symptoms to primary care in a low-income setting.
- Antibiotic exposure was assessed using two different methods

For peer review only

- Testing for sexually transmitted infections was limited to three major pathogens which may have led to an underestimation of the prevalence of sexually transmitted infections.
- The prevalence of sexually transmitted infections is not generalisable to all primary health care attendees, as the participants were enrolled if they reported the presence of urinary tract infection symptoms.

For peer review only

For peer review only - http://bmjopen.bmj.com/site/about/guidelines.xhtml

Introduction

Urinary tract infections (UTIs) are a common reason for presentation to primary care with 10% of adult women experiencing at least one episode per year.[1] Dysuria, which is one of the most common symptoms suggestive of a UTI, is reported in 2-5% of presentations in general practice.[2] While dysuria and other urinary symptoms such as frequency and urgency may be attributed to acute bacterial cystitis, they are by no means specific.[3] Symptoms can be due to a wide range of infectious agents as well as to other non-infectious causes (Table 1). In high-income settings acute bacterial cystitis is the most common cause of UTI symptoms in women presenting to primary care.[2] Thus the symptom complex is treated empirically with antibiotics and diagnostic tests are not deemed routinely necessary. Patients whose symptoms do not respond to first-line antibiotics are reassessed for other diagnoses or infections with organisms resistant to first-line antibiotics.[4]

Table 1. Causes of symptoms with acute onset that may be suggestive of a urinary tract infection in adults

**Category**		**Specific cause**
**Infectious**	Acute bacterial cystitis	*Escherichia coli*<br>*Klebsiella pneumoniae*<br>*Staphylococcus saprophyticus*<br>*Enterococcus spp.*<br>Unidentified (partially treated)
	Prostatitis	*Escherichia coli*<br>*Klebsiella pneumoniae*<br>*Proteus mirabilis*<br>*Pseudomonas aeruginosa*<br>*Enterococcus spp.*
	Sexually transmitted infections	*Chlamydia trachomatis*<br>*Neisseria gonorrhoea*<br>*Trichomonas vaginalis*<br>*Mycoplasma hominis*<br>*Ureaplasma urealyticum*<br>*Herpes simplex*<br>*Candida spp.*
	Other infections	*Mycobacterium tuberculosis* (urogenital)<br>*Schistosoma haematobium*
**Inflammatory**	Dermatologic	Irritant or contact dermatitis<br>Stevens-Johnson syndrome
	Non-infectious	Foreign body (e.g. stone)<br>Reactive arthritis with urethritis
**Non-inflammatory**	Drug or food related	Spermicides, topical hygiene products, drugs, certain foods
	Traumatic	Urogenital instrumentation/ surgery<br>Horseback or bicycle riding

For peer review only

The prevalence of positive urine cultures in patients presenting with presumed UTIs varies widely across different settings. This is particularly true when comparing studies from high- and low-income settings.[2, 5] Possible explanations include i) differences in prevalence of other conditions causing similar symptoms; ii) unregulated access to antibiotics leading to partially treated infections; or to iii) differences in laboratory techniques affecting the limit of detection.

This study aimed to evaluate possible causes of negative urine cultures in patients presenting with symptoms of UTI to primary health clinics in Harare.

Methods

Adults (aged 18 years and above) presenting with symptoms of UTIs to nine primary health clinics in Harare were enrolled into the ARGUS study (Antimicrobial Resistance in Gram-negatives from Urinary Specimens). In Zimbabwe, primary health clinics are nurse-led and serve the population living in their catchment area. Patients accessing the acute medical services have to pay a user fee. Access to diagnostic tests is limited beyond HIV, tuberculosis and malaria. The eligibility criteria and enrolment procedures for the ARGUS study were previously described.[6] Patients were included into the study if they presented with at least two symptoms suggestive of UTI (dysuria, urgency, frequency, suprapubic pain and/or flank pain).

This is a cross-sectional study reporting on a subset of participants from the ARGUS study who were consecutively enrolled between March and July 2020. Mid-stream urine samples were collected from study participants and underwent dipstick testing, microscopy and culture using conventional microbiology methods.[6] Leukocyturia was considered to be present if the sample had ≥10 white blood cells/µl on microscopy or a positive dipstick for leucocytes. Antibacterial activity of urine indicating possible prior antibiotic exposure was ascertained using a urine bioassay. Briefly, 20 µl urine were inoculated on a 6-mm filter paper disc that was incubated on Muller-Hinton and using *E. coli (*ATCC25922) as an indicator organism.[7] Antibacterial activity was considered to be present in the sample if a growth inhibition zone of any size around the disc was observed following incubation for 24 hours at 37°C.

Urine samples were tested for sexually transmitted infections (STIs), specifically *Chlamydia trachomatis* and *Neisseria gonorrhoea*. For women only, urine samples were also tested for

For peer review only - http://bmjopen.bmj.com/site/about/guidelines.xhtml

Trichomonas vaginalis. STI testing was done using the GeneXpert platform (Cepheid, Sunnyvale, CA) according to the manufacturer's instructions. Treatment for STIs was prescribed during the initial visit by the clinic nurse according to routine practice following the Zimbabwean national guidelines [8] and independent of study procedures. Participants with positive tests were notified of their result and offered treatment for themselves and their partners if they were not treated on their initial appointment. To ascertain if symptoms had resolved, a follow-up phone call was conducted 28 days post enrolment.

Statistical analysis

Data were analysed using STATA v.15 (StataCorp, TX, USA). Categorical data were described using counts and percentages and continuous data using medians and interquartile ranges (IQRs). Differences between categorical and continuous variables were evaluated using the chi-square and Mann Whitney U tests, respectively. The level of significance was set at $p \leq 0.05$.

The study was approved by the London School of Hygiene and Tropical Medicine ethics committee (Ref. 16424) and the Medical Research Council of Zimbabwe (MRCZ/A/2406). All study participants provided written informed consent.

Patient and public involvement

None in the study design. Results from this study were disseminated to nurses working in the polyclinics from where the study participants were recruited in order to improve patient care.

Results

The study included 425 participants with a median age of 37.3 years (IQR 26.8-48.0), of whom 275 (64.7%) were women and 172 (40.5%) were HIV-positive. All samples were tested for the presence of leukocytes, underwent conventional urine culture and were tested for *C. trachomatis* and *N. gonorrhoea*. Samples from 175 women were tested for *T. vaginalis*. Of 425 urine samples collected, 420 were stored for the antibiotic bioassay; volume was insufficient for storage for the remaining five samples. In 255 (60.0%) of participants at least one of the tests was positive while in 170 (40.0%) all of the tests that were performed were negative and the cause of their symptoms could not be determined or suspected. Figure 1 shows the distribution of positive test results [STI positive 83 (19.6%), urine culture positive 96 (22.6%), leukocyturia 130 (30.6%) and prior antibiotics 89 (20.9%)]. Haematuria was detected in 2 (0.5%) participants.

For peer review only - http://bmjopen.bmj.com/site/about/guidelines.xhtml

Urine cultures were positive in 96 (22.6%), negative in 314 (73.9%) and contaminated in 15 (3.5%) participants. A higher proportion of urine cultures were positive in women (n=73; 26.6%) compared to men (n=23, 15.3%). Urine culture positivity was higher in participants with more recent symptom onset prior to presentation (58/225, 25.8% in those with a symptom onset of ≤7 days, vs. 37/197, 18.8% for those with symptoms >7 days, p=0.086).

Overall, 83/425 (19.5%, 95%CI 15.9-23.6) participants had an STI including 11 who also had a positive urine culture. The prevalence of *C. trachomatis* was 43/425 (10.1%, 95%CI 7.4-13.4%), of *N. gonorrhoeae* 37/425 (8.7%, 95%CI 6.2-11.8) and of *T. vaginalis* 14/175 (8.0%, 95%CI 4.4-13.1). Eleven (2.6%) participants had a positive STI test for two pathogens. The prevalence of *C. trachomatis* was 26/275 (9.5% 95%CI 6.3-13.5) among women and 17/133 (12.8% 95%CI 7.6-19.7) among men. For *N. gonorrhoea* prevalence was 18/275 (6.5% 95%CI 3.6-10.1) among women and 19/150 (12.7% 95%CI 7.8-19.1, p=0.032) among men. STIs were more common in those with recent symptom onset (52/225, 23.1% among those with an onset of symptoms of ≤7 days and 31/197, 15.7% for those with symptoms >7 days, p =0.057). Participants who tested positive for an STI, were younger and were less likely to have HIV-infection than those without an STI (Table 2).

Table 2. Characteristics of study participants according to test result

Characteristic	Total N=425 (%)	STI positive N=83 (%)	Urine culture positive N=96 (%)	Leukocyturia N=130 (%)	Prior antibiotics N=89 (%)	All tests negative N=170 (%)
Age, median (IQR)	37.3 (26.8-48.0)	28.4 (23.5-39.2)	39.8 (26.0-50.0)	34.2 (24.8-47.0)	37.6 (27.6-49.4)	39.4 (29.6-49.6)
Female sex	275 (64.7)	51 (61.5)	73 (76.0)	88 (67.7)	51 (57.3)	107 (62.9)
Completed at least secondary education	359 (84.5)	71 (85.5)	75 (78.1)	108 (83.1)	77 (86.5)	141 (82.9)
HIV positive*	172 (42.4)	20 (25.6)	42 (47.2)	53 (43.4)	38 (44.2)	76 (46.1)
Pregnancy*	35 (13.0)	4 (7.8)	7 (9.7)	15 (17.2)	5 (9.8)	16 (15.5)
Married/ have a partner	325 (76.5)	66 (79.5)	74 (77.1)	91 (70.0)	67 (75.3)	132 (77.7)
Duration of symptoms prior to presentation	7 (5-12)	7 (4-12)	7 (4-11)	7 (5-10)	7 (5-12)	9 (6-12)
Symptoms resolved on follow-up*	334 (85.0)	70 (92.1)	74 (83.2)	105 (89.0)	73 (89.0)	127 (79.9)

For peer review only - http://bmjopen.bmj.com/site/about/guidelines.xhtml

**19 participants did not know their HIV status; 5 women did not know if they were pregnant; follow-up information available for 393 participants.*
IQR: interquartile range; STI: sexually transmitted infection

The median duration of symptoms was 7 days for participants with positive urine cultures and those who had an STI. Among participants who did not have an STI or a positive urine culture, the median duration of symptoms was 8 days (IQR 5-12). The most common presenting symptoms were dysuria (337/425, 79.3%), frequency (310/425, 72.9%), and suprapubic pain (272/425, 64.0%). Overall presenting symptoms were similar between individuals with STIs, UTIs and those with negative cultures (Figure 2).

Antimicrobial treatment prior to clinic presentation was reported by 30 (7.1%) while the antibiotic bioassay was positive in 73 (17.4%) including 26 participants with HIV infection who were taking co-trimoxazole prophylaxis. Overall, 89 (20.9%) of participants had either reported taking antibiotics or had a positive urine bioassay.

STI treatment was prescribed by the clinic nurses at the initial visit in 38 participants, of whom 20 (53%) had a positive STI test while 63 (76%) of the 83 patients who had an STI were not prescribed STI treatment by the primary health care nurse.

A follow up phone call was made with 393 (92.5%) of the participants. Most (334/393, 85.0%) reported that the symptoms had resolved or significantly improved since enrolment. Among the 59 participants whose symptoms did not improve or resolve, 15 had a positive urine culture and six had an STI while 32 (54.2%) did not have any positive tests. Symptom resolution or improvement was reported by 70/76 (92.1%) participants with an STI, by 74/89 (83.1%) of those with a positive urine culture and by 127/159 (79.9%) with negative results in all tests. Among participants with positive urine cultures, symptoms improved or resolved in 65/69 (94.2%) of those who reported having taken their prescribed treatment and in 9/20 (45%) of those who had not.

Discussion

This study found that only one in five people presenting to primary care in Harare with UTI symptoms had a positive urine culture with a uropathogen explaining their presenting symptoms. There was a high prevalence of STIs and evidence of prior antimicrobial use among

For peer review only - http://bmjopen.bmj.com/site/about/guidelines.xhtml

study participants as possible explanations for the low proportion of positive urine cultures. However, in 40% of patients, symptoms remained unexplained.

Only 20% of study participants had a positive urine culture which is similar to findings from studies conducted in other sub-Saharan African countries where culture positivity was found to be 11% and 36% among outpatients.[5, 9] In contrast, studies conducted among women with cystitis in high-income countries mostly from Europe and the Americas, report that 75-80% of urine cultures reveal significant growth of uropathogens.[2, 10, 11] This may be explained by differences in access to healthcare, unregulated antibiotic use and prevalence of other conditions with similar symptoms between settings.[5, 12-14]

In our study, the median time to presentation was 7 days while studies from high-income settings report a much shorter time between symptom onset and clinic presentation of 2-3 days.[10, 11] Thus, the lower yield of urine cultures in this study may be due to spontaneous resolution of infection because of the delays in accessing healthcare or self-treatment with antibiotics.[15] This may particularly be the case in patients with leukocyturia where symptoms can be explained by residual inflammation. Dysuria and leukocyturia in the absence of positive urine cultures may be due to other causes such as urogenital tuberculosis, schistosomiasis and other inflammatory conditions.[3] However, other infections are unlikely to be the cause of symptoms in our patient population given that for the vast majority, the symptoms resolved or improved on follow-up and that haematuria was present in less than 1%.

Antimicrobial use prior to clinic presentation may also have contributed to a lower urine culture yield with one in five study participants having evidence of taking antibiotics prior to presentation to primary care. While only 7% of the study participants reported antimicrobial use in the previous two weeks, 17% had evidence of antimicrobial activity in their urine. Although in Zimbabwe antibiotic access from pharmacies is highly regulated, antibiotics can be acquired from the informal market.[16] Patients may not report on self-treatment at clinic presentation due to social desirability bias. Prior antimicrobial use was similar to that reported by patients from Senegal [17] which had a culture positivity of 27% but lower than in studies from the United States.[18] This suggests that asking patients about antimicrobials may not be sufficient in determining prior exposure as shown in findings from Ghana.[19] On the other hand the antibiotic bioassay may overestimate antimicrobial use for treatment of infections by also detecting antimicrobials consumed unintentionally in food and water.[20] Alternative

For peer review only

explanations for symptoms suggestive of UTIs such as dysuria include irritant hygienic products, spermicides and certain foods.[3]

STIs are highly prevalent in some sub-Saharan African setting[21] and there is some symptom overlap between STIs and UTIs. A meta-analysis of studies from sub-Saharan Africa reported a pooled prevalence for *C. trachomatis* of 7.8% in women of reproductive age[22] while a study from Zimbabwe testing mainly asymptomatic adolescents and young people found a prevalence of 17% of *C. trachomatis* and/or *Neisseria gonorrhoea*.[23] While STIs may be asymptomatic particularly in women, patients with symptoms due to STIs frequently experience diagnostic and treatment delays leading to prolonged symptoms prior to presentation.[24] One of the study eligibility criteria included recent symptom onset (within two weeks). This was aimed at increasing the likelihood of UTIs compared to STIs. Despite this, STI prevalence in our study was almost 20%. Importantly one in fifteen women and one in eight men tested positive for *Neisseria gonorrhoea*. The high prevalence of STIs may also explain the large proportion of men enrolled into the study, who are generally more likely to experience symptoms from STIs, which are similar to symptoms of UTIs.[24] The study may have missed other STIs because testing was limited to three major pathogens but did not include other organisms that may cause urethritis and urinary tract symptoms such as *Mycoplasma genitalium* leading to an underestimation of STI prevalence. Furthermore, STIs may have been missed because the study used mid-stream urine samples while first-catch samples are recommended for the diagnosis of STIs for optimal yield on urine samples. Genital ulcers caused by herpes simplex and vaginitis due to candida may explain symptoms in some patients. Also, men were not tested for *T. vaginalis* as this is less frequent in men.[25] Potential participants who did not seek care because of not being able to afford the clinic fees would have been missed.

Only a quarter of participants who tested positive for an STI, received STI treatment on the initial clinic presentation because the attending nurse did not suspect an STI. This highlights the difficulties in differentiating the two conditions on clinical grounds. Access to diagnostic testing at primary care level is thus urgently needed.

The prevalence of STIs is not generalisable to all primary health care attendees, as the participants were selected on the basis of STI/UTI symptoms.

For peer review only - http://bmjopen.bmj.com/site/about/guidelines.xhtml

Conclusions

This is the first study exploring the causes for negative urine cultures among adults presenting with UTI symptoms to primary care in a low-income setting. The findings of this study highlight the high STI burden in this patient population. The overlap in symptoms in patients diagnosed with UTIs and STIs respectively results in underdiagnosis and undertreatment of STIs. Diagnostic testing is paramount to ensure appropriate treatment. Very few participants who had an STI received appropriate treatment on clinic presentation because STIs were not suspected emphasizing the missed opportunities for diagnosis and treatment. The high prevalence of prior antibiotic exposure in patients presenting to primary care should prompt an improvement of regulations around antibiotic access to prevent development of antimicrobial resistance.

Funding

IDO received funding though the Wellcome Trust Clinical PhD Programme awarded to the London School of Hygiene & Tropical Medicine (grant number 203905/Z/16/Z). The funders had no role in study design, data collection and interpretation, or the decision to submit the work for publication. The study was funded by UK aid from the UK government; the views expressed, however, do not necessarily reflect the UK government's official policies.

Competing interests: none declared.

Ethical approval: The study was approved by the London School of Hygiene and Tropical Medicine ethics committee (Ref. 16424) and the Medical Research Council of Zimbabwe (MRCZ/A/2406). All study participants provided written informed consent.

Data availability statement: the data underlying this study can be obtained upon request by emailing the corresponding author.

Author contributions: IDO and KK conceived the study idea and planned the study. RAF contributed to the study idea. PC, KPEM and AM provided support with the resources needed for the study. SY and RAF provided support with conducting the study. IDO and MC conducted the laboratory testing and interpretation of results. IDO and KK analysed and interpreted the data, and drafted the initial manuscript. MC, SY, DM, MM, PC, KPEM, AM, and RAF reviewed and provided feedback on the initial manuscript. All authors read and approved the final manuscript.

For peer review only - http://bmjopen.bmj.com/site/about/guidelines.xhtml

For peer review only

For peer review only - http://bmjopen.bmj.com/site/about/guidelines.xhtml

Figure 1. Distribution of positive tests among study participants (170/425 samples, 40% were negative for all tests)

Figure 2. Presenting symptoms in study participants according to urine culture and STI test result

Severe symptoms were defined as symptoms affecting daily activities

For peer review only

References

1. Foxman B, Barlow R, D'Arcy H, Gillespie B, Sobel JD. Urinary tract infection: self-reported incidence and associated costs. Ann Epidemiol. 2000;10(8):509-15. Epub 2000/12/19. doi: 10.1016/s1047-2797(00)00072-7. PubMed PMID: 11118930.
2. Heytens S, Boelens J, Claeys G, DeSutter A, Christiaens T. Uropathogen distribution and antimicrobial susceptibility in uncomplicated cystitis in Belgium, a high antibiotics prescribing country: 20-year surveillance. Eur J Clin Microbiol Infect Dis. 2017;36(1):105-13. Epub 2016/09/19. doi: 10.1007/s10096-016-2776-8. PubMed PMID: 27639858.
3. Michels TC, Sands JE. Dysuria: Evaluation and Differential Diagnosis in Adults. Am Fam Physician. 2015;92(9):778-86. Epub 2015/11/12. PubMed PMID: 26554471.
4. World Health Organization. IMAI district clinician manual: hospital care for adolescents and adults: guidelines for the management

of illnesses with limited-resources. WHO, Geneva, 2011. Available from https://apps.who.int/iris/bitstream/handle/10665/77751/9789241548281_Vol1_eng.pdf.
5. Iregbu K.C., P.I. N-P. Urinary tract infections in a tertiary hospital in Abuja, Nigeria. African Journal of Clinical and Experimental Microbiology. 2013;14:169-73.
6. Olaru ID, Yeung S, Ferrand RA, Stabler R, Chonzi P, Mabey D, et al. Antimicrobial Resistance in Gram-negative bacteria from Urinary Specimens: a study of prevalence, risk factors and molecular mechanisms of resistance (ARGUS) in Zimbabwe - a study protocol. Wellcome Open Res. 2020;5:140. Epub 2020/10/23. doi: 10.12688/wellcomeopenres.15977.1. PubMed PMID: 33088923; PubMed Central PMCID: PMCPMC7551514.
7. Driscoll AJ, Bhat N, Karron RA, O'Brien KL, Murdoch DR. Disk diffusion bioassays for the detection of antibiotic activity in body fluids: applications for the Pneumonia Etiology Research for Child Health project. Clin Infect Dis. 2012;54 Suppl 2:S159-64. Epub 2012/03/21. doi: 10.1093/cid/cir1061. PubMed PMID: 22403231.
8. EDLIZ 2015 - 7th Essential Medicines List and Standard Treatment Guidelines for Zimbabwe. The National Medicine and Therapeutics Policy Advisory Committee [NMTPAC], Ministry of Health & Child Welfare, Republic of Zimbabwe.
9. Bitew A, Molalign T, Chanie M. Species distribution and antibiotic susceptibility profile of bacterial uropathogens among patients complaining urinary tract infections. BMC Infect Dis. 2017;17(1):654. Epub 2017/10/01. doi: 10.1186/s12879-017-2743-8. PubMed PMID: 28962545; PubMed Central PMCID: PMCPMC5622472.
10. Hooton TM, Roberts PL, Cox ME, Stapleton AE. Voided midstream urine culture and acute cystitis in premenopausal women. N Engl J Med. 2013;369(20):1883-91. Epub 2013/11/15. doi: 10.1056/NEJMoa1302186. PubMed PMID: 24224622; PubMed Central PMCID: PMCPMC4041367.
11. Naber KG, Schito G, Botto H, Palou J, Mazzei T. Surveillance study in Europe and Brazil on clinical aspects and Antimicrobial Resistance Epidemiology in Females with Cystitis (ARESC): implications for empiric therapy. Eur Urol. 2008;54(5):1164-75. Epub 2008/05/31. doi: 10.1016/j.eururo.2008.05.010. PubMed PMID: 18511178.
12. Auta A, Hadi MA, Oga E, Adewuyi EO, Abdu-Aguye SN, Adeloye D, et al. Global access to antibiotics without prescription in community pharmacies: A systematic review and meta-analysis. J Infect. 2019;78(1):8-18. Epub 2018/07/10. doi: 10.1016/j.jinf.2018.07.001. PubMed PMID: 29981773.
13. Bichler KH, Savatovsky I, Members of the Urinary Tract Infection Working Group of the Guidelines Office of the European Association of U, Naber KG, Bischop MC, Bjerklund-Johansen TE, et al. EAU guidelines for the management of urogenital schistosomiasis. Eur Urol. 2006;49(6):998-1003. Epub 2006/03/08. doi: 10.1016/j.eururo.2006.02.022. PubMed PMID: 16519990.
14. Muneer A, Macrae B, Krishnamoorthy S, Zumla A. Urogenital tuberculosis - epidemiology, pathogenesis and clinical features. Nat Rev Urol. 2019;16(10):573-98. Epub 2019/09/25. doi: 10.1038/s41585-019-0228-9. PubMed PMID: 31548730.

For peer review only - http://bmjopen.bmj.com/site/about/guidelines.xhtml

15. Hoffmann T, Peiris R, Mar CD, Cleo G, Glasziou P. Natural history of uncomplicated urinary tract infection without antibiotics: a systematic review. Br J Gen Pract. 2020;70(699):e714-e22. Epub 2020/09/23. doi: 10.3399/bjgp20X712781. PubMed PMID: 32958533; PubMed Central PMCID: PMCPMC7510849.
16. EUCAST guidelines for detection of resistance mechanisms and specific resistances of clinical and/or epidemiological importance Version 2.01 July 2017. Available from https://eucast.org/resistance_mechanisms/.
17. Barry MS, Diallo BA, Kante D, Diallo IS. Antimicrobial susceptibility profile of community-acquired urinary tract infection in adults: A seven months prospective cross-sectional study in Dakar Town, Senegal. African Journal of Urology. 2017;23(2):166-71.
18. Talan DA, Krishnadasan A, Abrahamian FM, Stamm WE, Moran GJ, Group EMINS. Prevalence and risk factor analysis of trimethoprim-sulfamethoxazole- and fluoroquinolone-resistant Escherichia coli infection among emergency department patients with pyelonephritis. Clin Infect Dis. 2008;47(9):1150-8. Epub 2008/09/24. doi: 10.1086/592250. PubMed PMID: 18808361.
19. Donkor GY, Dontoh E, Owusu-Ofori A. A cross-sectional study on the prevalence of antibiotic use prior to laboratory tests at two Ghanaian hospitals. PLoS One. 2019;14(1):e0210716. Epub 2019/01/16. doi: 10.1371/journal.pone.0210716. PubMed PMID: 30645626; PubMed Central PMCID: PMCPMC6333348.
20. Azanu D, Styrishave B, Darko G, Weisser JJ, Abaidoo RC. Occurrence and risk assessment of antibiotics in water and lettuce in Ghana. Sci Total Environ. 2018;622-623:293-305. Epub 2017/12/08. doi: 10.1016/j.scitotenv.2017.11.287. PubMed PMID: 29216470.
21. Rowley J, Vander Hoorn S, Korenromp E, Low N, Unemo M, Abu-Raddad LJ, et al. Chlamydia, gonorrhoea, trichomoniasis and syphilis: global prevalence and incidence estimates, 2016. Bull World Health Organ. 2019;97(8):548-62P. Epub 2019/08/07. doi: 10.2471/BLT.18.228486. PubMed PMID: 31384073; PubMed Central PMCID: PMCPMC6653813.
22. Hussen S, Wachamo D, Yohannes Z, Tadesse E. Prevalence of chlamydia trachomatis infection among reproductive age women in sub Saharan Africa: a systematic review and meta-analysis. BMC Infect Dis. 2018;18(1):596. Epub 2018/11/28. doi: 10.1186/s12879-018-3477-y. PubMed PMID: 30477441; PubMed Central PMCID: PMCPMC6258386.
23. Martin K, Olaru ID, Buwu N, Bandason T, Marks M, Dauya E, et al. Uptake of and factors associated with testing for sexually transmitted infections in community-based settings among youth in Zimbabwe: a mixed-methods study. Lancet Child Adolesc Health. 2021;5(2):122-32. Epub 2021/01/09. doi: 10.1016/S2352-4642(20)30335-7. PubMed PMID: 33417838.
24. Hoffman CM, Fritz L, Matlakala N, Mbambazela N, Railton JP, McIntyre JA, et al. Community-based strategies to identify the unmet need for care of individuals with sexually transmitted infection-associated symptoms in rural South Africa. Trop Med Int Health. 2019;24(8):987-93. Epub 2019/05/30. doi: 10.1111/tmi.13274. PubMed PMID: 31141301.
25. Chitneni P, Beksinska M, Dietrich JJ, Jaggernath M, Closson K, Smith P, et al. Partner notification and treatment outcomes among South African adolescents and young adults diagnosed with a sexually transmitted infection via laboratory-based screening. Int J STD AIDS. 2020;31(7):627-36. Epub 2020/05/15. doi: 10.1177/0956462420915395. PubMed PMID: 32403988; PubMed Central PMCID: PMCPMC7357572.

For peer review only - http://bmjopen.bmj.com/site/about/guidelines.xhtml

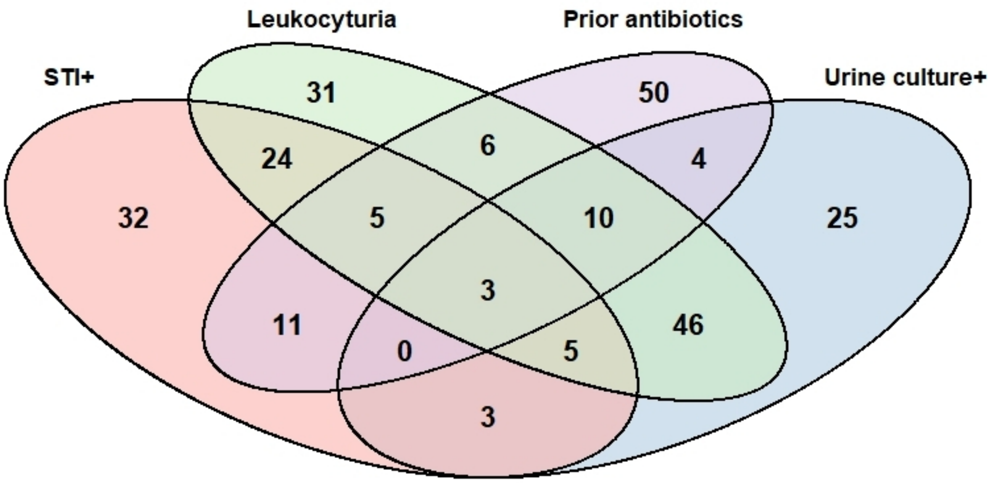

64x38mm (300 x 300 DPI)

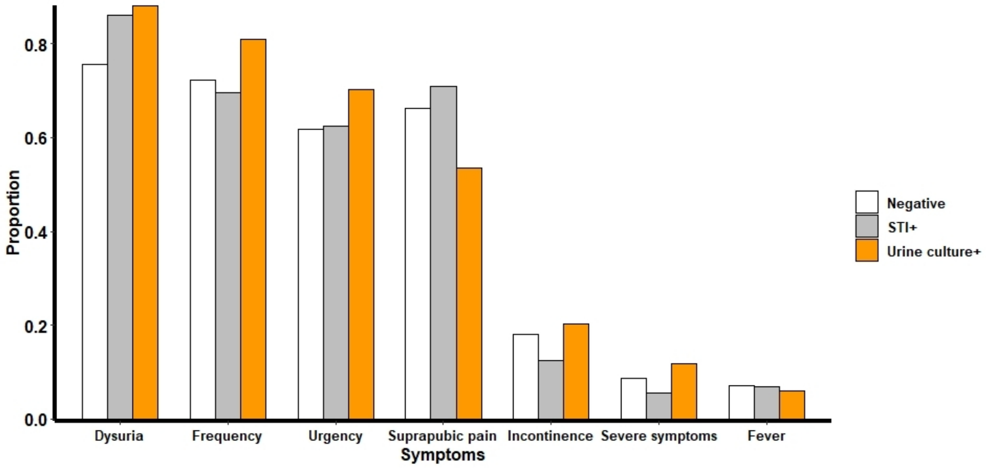

80x38mm (300 x 300 DPI)

STROBE Statement—Checklist of items that should be included in reports of ***cross-sectional studies***

	Item No	Recommendation	Page No
**Title and abstract**	1	(*a*) Indicate the study's design with a commonly used term in the title or the abstract	2
		(*b*) Provide in the abstract an informative and balanced summary of what was done and what was found	2
**Introduction**			
Background/rationale	2	Explain the scientific background and rationale for the investigation being reported	3-4
Objectives	3	State specific objectives, including any prespecified hypotheses	4
**Methods**			
Study design	4	Present key elements of study design early in the paper	4
Setting	5	Describe the setting, locations, and relevant dates, including periods of recruitment, exposure, follow-up, and data collection	4-5
Participants	6	(*a*) Give the eligibility criteria, and the sources and methods of selection of participants	4
Variables	7	Clearly define all outcomes, exposures, predictors, potential confounders, and effect modifiers. Give diagnostic criteria, if applicable	4-5
Data sources/ measurement	8*	For each variable of interest, give sources of data and details of methods of assessment (measurement). Describe comparability of assessment methods if there is more than one group	4-5
Bias	9	Describe any efforts to address potential sources of bias	4-5
Study size	10	Explain how the study size was arrived at	4
Quantitative variables	11	Explain how quantitative variables were handled in the analyses. If applicable, describe which groupings were chosen and why	5
Statistical methods	12	(*a*) Describe all statistical methods, including those used to control for confounding	5
		(*b*) Describe any methods used to examine subgroups and interactions	NA
		(*c*) Explain how missing data were addressed	4-5
		(*d*) If applicable, describe analytical methods taking account of sampling strategy	NA
		(*e*) Describe any sensitivity analyses	NA
**Results**			
Participants	13*	(a) Report numbers of individuals at each stage of study—eg numbers potentially eligible, examined for eligibility, confirmed eligible, included in the study, completing follow-up, and analysed	4-5
		(b) Give reasons for non-participation at each stage	NA
		(c) Consider use of a flow diagram	NA
Descriptive data	14*	(a) Give characteristics of study participants (eg demographic, clinical, social) and information on exposures and potential confounders	5
		(b) Indicate number of participants with missing data for each variable of interest	5-6
Outcome data	15*	Report numbers of outcome events or summary measures	5-7
Main results	16	(*a*) Give unadjusted estimates and, if applicable, confounder-adjusted estimates and their precision (eg, 95% confidence interval). Make clear which confounders were adjusted for and why they were included	5-7

For peer review only - http://bmjopen.bmj.com/site/about/guidelines.xhtml

		(*b*) Report category boundaries when continuous variables were categorized	NA
		(*c*) If relevant, consider translating estimates of relative risk into absolute risk for a meaningful time period	NA
Other analyses	17	Report other analyses done—eg analyses of subgroups and interactions, and sensitivity analyses	5-7
**Discussion**			
Key results	18	Summarise key results with reference to study objectives	7
Limitations	19	Discuss limitations of the study, taking into account sources of potential bias or imprecision. Discuss both direction and magnitude of any potential bias	9
Interpretation	20	Give a cautious overall interpretation of results considering objectives, limitations, multiplicity of analyses, results from similar studies, and other relevant evidence	7-9
Generalisability	21	Discuss the generalisability (external validity) of the study results	9
**Other information**			
Funding	22	Give the source of funding and the role of the funders for the present study and, if applicable, for the original study on which the present article is based	10

*Give information separately for exposed and unexposed groups.

Note: An Explanation and Elaboration article discusses each checklist item and gives methodological background and published examples of transparent reporting. The STROBE checklist is best used in conjunction with this article (freely available on the Web sites of PLoS Medicine at http://www.plosmedicine.org/, Annals of Internal Medicine at http://www.annals.org/, and Epidemiology at http://www.epidem.com/). Information on the STROBE Initiative is available at www.strobe-statement.org.

For peer review only
